# Supplementary material for: Learning curve and surgical outcome of robotic assisted colorectal surgery with ERAS program
Source: Sci Rep. 2022 Nov 29;12:20566. doi: 10.1038/s41598-022-24665-w (PMC9709162; doi:10.1038/s41598-022-24665-w)
Supplement: Supplementary file 1 — Supplementary Information 1. [file 41598_2022_24665_MOESM1_ESM.zip › LSC Raw data-Ñ[▒K/050/P_ñw╝╨░O▒Kñσ.pdf]

[redacted]

Pathologic diagnosis:
1. Rectum, TaTME --- a. Status post CCRT with no residual tumor. The bilateral cut ends and mesocolic lymph nodes (0/7) are free of tumor.
b. Hyperplastic polyps.
2. IMA root, dissection --- Unremarkable.
3. Lymph node, right pelvic, dissection --- Unremarkable (0/2).

Prognostic and predictive factor:
1. Tumor size: Cannot be assessed.
2. Depth of invasion: No evidence of primary tumor.
3. Histologic grade: GX: Cannot be assessed.
4. Angiolymphatic invasion: Not identified.
5. Perineural invasion: Not identified.
6. Tumor deposits (discontinuous extramural extension): Not identified.
7. Surgical Margins: a) Proximal Margin: Uninvolved, distance of stitch from margin: 14 cm. b) Distal Margin: Uninvolved, distance of stitch from margin: 1 cm. c) Radial or Mesenteric Margin: Uninvolved by invasive carcinoma, distance of stitch from margin: 15 cm.
8. Lymph node status: Uninvolved (0/7).
9. Extranodal involvement: Not applicable.
10. Tumor regression grading S/P CCRT: Present, no viable cancer cells (complete response, score 0).
11. Type of polyp in which invasive carcinoma arose: Not applicable.
12. Additional pathologic findings: Hyperplastic polyp.
13. Pathological TNM Stage: ypTxN0(According to the eighth edition, 2017, American Joint Committee on Cancer Staging Guidelines for Tumors / 2017 CAP guideline).
14. TNM descriptors: y (Post-treatment).

Gross description:
The specimen consists of 1) a segment of rectum, 15 cm in length and 5 cm in proximal cut end circumference, 6 cm in distal circumference, and perirectal soft tissue. A stitch is noted 1 cm above the nearest cut end. No tumor is seen grossly on cut. The lymph node is hard to find grossly. 2) a piece of tan gray soft tissue, 3.5x2x1 cm, labeled as "IMA root". 3) a piece of tan gray soft tissue, 6.5x3x0.4 cm, labeled as "right pelvic lymph node". All for section: A) specimen 2; Representative part for section: B) specimen 3 C) proximal cut end D) distal cut end E) cut end F-J) mucosa near the stitch K) polyps L-V) lymph node.
#T-59600_2 #M-09183_2 0 1105 00SP00
#T-C4520_2 #M-09183_2 0 1105 000000

已電子簽章

敬告：本單僅供醫師參考使用，不具任何申請效力！！
請病患妥為保管，若不慎遺失需自負個人資料外洩責任！
